# A machine learning-based radiomics approach for differentiating patellofemoral osteoarthritis from non-patellofemoral osteoarthritis using Q-Dixon MRI

**DOI:** 10.3389/fspor.2025.1535519

**Published:** 2025-01-17

**Authors:** Liangjing Lyu, Jing Ren, Wenjie Lu, Jingyu Zhong, Yang Song, Yongliang Li, Weiwu Yao

**Affiliations:** ^1^ Department of Radiology, Tongren Hospital, Shanghai Jiao Tong University School of Medicine, Shanghai, China; ^2^ MR Research Collaboration Team, Siemens Healthineers Ltd., Shanghai, China

**Keywords:** anterior knee pain, patellofemoral osteoarthritis, Q-Dixon MRI, radiomics, machine learning, fat fraction, quadriceps fat pad

## Abstract

This prospective diagnostic study aimed to assess the utility of machine learning-based quadriceps fat pad (QFP) radiomics in distinguishing patellofemoral osteoarthritis (PFOA) from non-PFOA using Q-Dixon MRI in patients presenting with anterior knee pain. This diagnostic accuracy study retrospectively analyzed data from 215 patients (mean age: 54.2 ± 11.3 years; 113 women). Three predictive models were evaluated: a proton density-weighted image model, a fat fraction model, and a merged model. Feature selection was conducted using analysis of variance, and logistic regression was applied for classification. Data were collected from training, internal, and external test cohorts. Radiomics features were extracted from Q-Dixon MRI sequences to distinguish PFOA from non-PFOA. The diagnostic performance of the three models was compared using the area under the curve (AUC) values analyzed with the Delong test. In the training set (109 patients) and internal test set (73 patients), the merged model exhibited optimal performance, with AUCs of 0.836 [95% confidence interval (CI): 0.762–0.910] and 0.826 (95% CI: 0.722–0.929), respectively. In the external test set (33 patients), the model achieved an AUC of 0.885 (95% CI: 0.768–1.000), with sensitivity and specificity values of 0.833 and 0.933, respectively (*p* < 0.001). Fat fraction features exhibited a stronger predictive value than shape-related features. Machine learning-based QFP radiomics using Q-Dixon MRI accurately distinguishes PFOA from non-PFOA, providing a non-invasive diagnostic approach for patients with anterior knee pain.

## 1 Introduction

Anterior knee pain (AKP) (1) is a common condition affecting approximately 40% of adolescent athletes (2). Young, active individuals with AKP seek care at sports injury clinics. A recent systematic review (3) highlighted that females have a two-fold higher risk of developing AKP due to patellofemoral issues compared with males. While AKP was previously considered a self-limiting condition, recent studies (4, 5) have demonstrated its potential to become chronic, often leading to psychological comorbidities. Patients with AKP experience reduced quality of life (6) and progressive patellofemoral cartilage damage (7). The etiology of AKP is multifaceted, involving multiple structures, including the patellofemoral joint and soft tissues, and thus requiring individualized treatment tailored to each patient's characteristics and the specific underlying cause. Patellofemoral osteoarthritis (PFOA) is more prevalent than tibiofemoral osteoarthritis (TFOA) (8), as it pertains to the flexion and extension movements of the knee joint rather than being associated with weight-bearing activities; however, it has received less attention. Additionally, the presence of PFOA increases the risk of OA in the tibiofemoral compartment (9). Simultaneously, early clinical intervention in the progression of PFOA can potentially maximize the avoidance of joint replacement outcomes (5). However, there has been a persistent lack of convenient and reliable quantitative methods for the assessment of PFOA.

Peripatellar fat pads and synovium function as a cohesive unit, with the fat pads playing a pivotal role in synovial inflammation, fibrosis, and osteoarthritis (OA)-associated pain (10, 11). The knee joint contains three types of fat pads: the quadriceps fat pad (QFP), also known as the suprapatellar fat pad; the prefemoral fat pad (PFP), which is separated from the QFP by the suprapatellar bursa; and the infrapatellar fat pad (IFP), also referred to as Hoffa's fat pad. These structures are integral to knee joint anatomy, contributing to its biomechanics, cushioning, and secretory functions (12, 13). The IFP has garnered significant attention. Edema of the IFP has shown strong correlations with knee OA (KOA), particularly TFOA (14–17). Recent radiomics studies (15, 18) have further underscored the diagnostic and therapeutic potential of the IFP in KOA, especially in TFOA. Similar to the IFP, the QFP and synovium function as a unit, playing a role in the early stages of synovial inflammation, fibrosis, and AKP (10). Conversely, research on the QFP in the context of KOA, and particularly PFOA, remains limited and has predominantly relied on conventional MRI sequences. MRI offers superior soft tissue resolution, enabling more sensitive detection of early pathological abnormalities in OA than radiography and CT imaging, which makes it a reliable tool in early intervention and monitoring treatment effects.

Despite its advantages, traditional MRI sequences cannot directly provide quantitative values and are limited in distinguishing the degree of edema in fat pads. Erber et al. (19) proposed a grading method for QFP edema based on conventional proton density-weighted image (PDWI) sequences. This method compares the signal of the QFP with those of the PFP and the gastrocnemius muscle. Grade A is defined by a QFP signal higher than that of the PFP but lower than that of the gastrocnemius, while a signal similar to that of the gastrocnemius is defined as Grade B, and one higher than that of the gastrocnemius is defined as Grade C. This classification method can only roughly divide the fat pad signal into four levels, which may cause difficulties in practice, especially when the signal of the fat pad is close to that of the gastrocnemius, or when there is also edema in the PFP, making visual assessment unreliable. Additionally, some studies (20, 21) focused on the mass effect of the QFP based on conventional PDWI, suspecting that the protruding morphological characteristics of the QFP might have certain clinical significance. However, only a rough visual assessment is possible, without precise quantitative analysis. Currently, the primary MRI sequences used for the measurement of fat content are Q-Dixon and proton magnetic resonance spectroscopy (MRS), both renowned for their robust accuracy and objectivity. Both techniques have been widely used in the quantification of fat in various tissues, including the liver, bones, and muscles (22, 23). However, the cubic volume-of-interest with a small volume of MRS restricts its clinical applications. Q-Dixon holds greater clinical potential since it provides scalable maps of fat distribution.The Q-Dixon MRI sequence employs a multi-echo, three-dimensional (3D) gradient echo volumetric interpolated breath-hold examination T2*-corrected 6-point Q-Dixon protocol, which is adept at generating images that distinguish between water, fat, T2*, R2*, in-phase, and opposed-phase signals. This enables the automated reconstruction of fat fraction (FF) maps, where the FF is defined as the ratio of the signal intensity from fat to the total signal intensity from both fat and water. Our previous research (24) has confirmed that the FF values obtained from Q-Dixon-based QFP are correlated with PFOA severity. Quantitative and radiomics data exploring the role of the QFP are sparse despite their relevance in PFOA processes. Furthermore, MRI sequences based on the Q-Dixon technology not only offer the advantage of a short scanning time of only 35 s but also provide a more comprehensive set of quantitative information compared with traditional MRI, indicating their significant potential in medical imaging.

We hypothesized that integrating the Q-Dixon technology with conventional MRI sequences would enhance the diagnostic performance of QFP radiomics, leading to improved diagnostic outcomes for PFOA. To test this hypothesis, this study aimed to evaluate the performance of QFP radiomic features derived from different sequences to serve as independent imaging markers for PFOA.

## 2 Materials and methods

### 2.1 Ethics approval and patient consent

This cross-sectional study with prospective data collection was approved by the Institutional Review Board of Shanghai Tongren Hospital (no. 2022-044-01) and adhered to the ethical standards outlined in the 2013 revision of the Declaration of Helsinki. Informed consent was obtained from all patients. This study followed the Strengthening the Reporting of Observational Studies in Epidemiology guidelines.

### 2.2 Study design and patients

**Figure 1:**
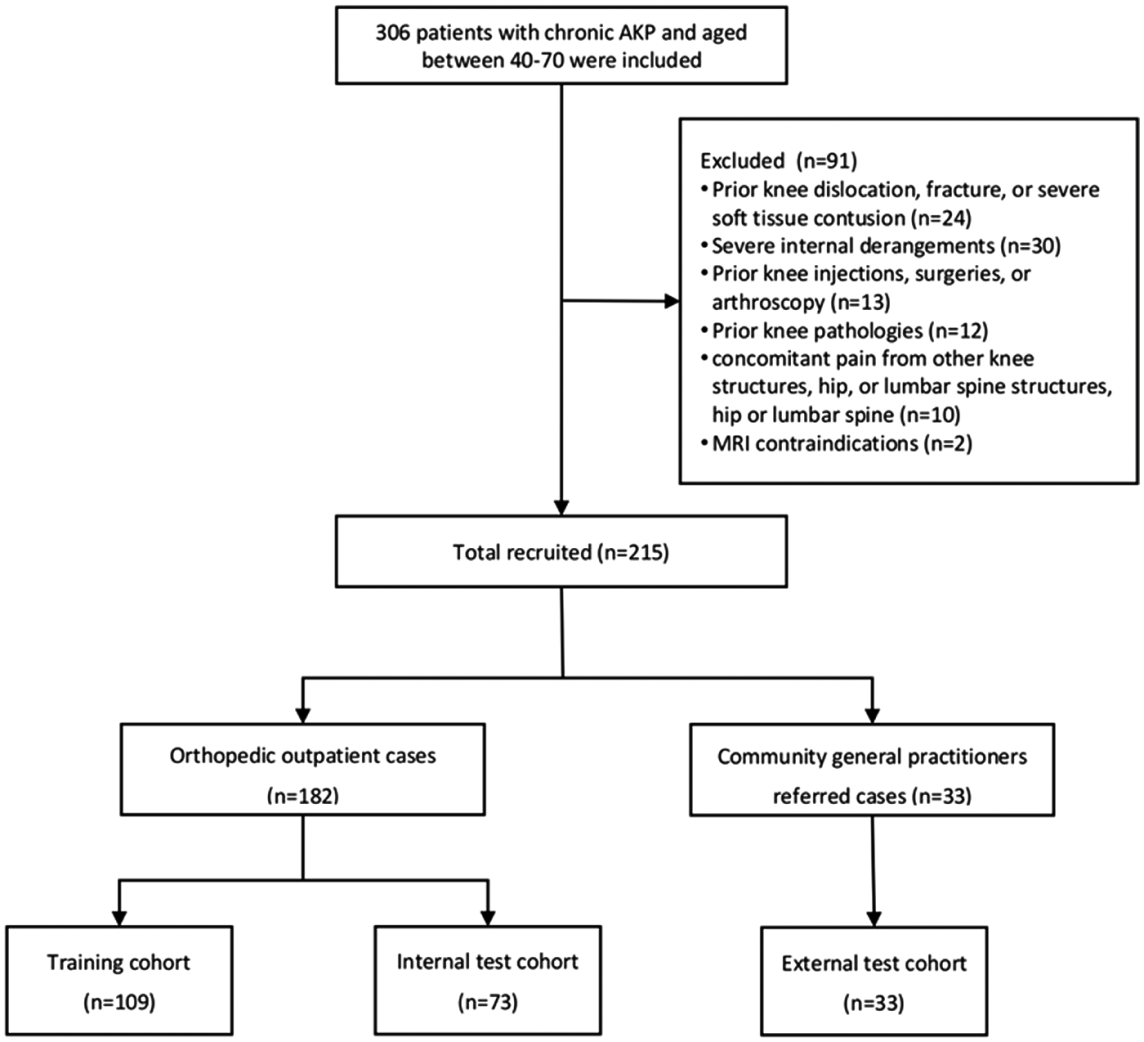
Patient selection flowchart.

A total of 306 patients diagnosed with AKP between August 1, 2023, and July 31, 2024, at orthopedic outpatient clinics and through referrals from community clinics were evaluated. The inclusion criteria included age 40–70 years and persistent pain in the patella region for more than two months. Exclusion criteria were as follows: (i) prior knee dislocation, fracture, or severe soft tissue contusion; (ii) severe internal derangements, such as meniscal, tendinous, or ligamentous tears; (iii) prior knee injections, surgeries, or arthroscopy; (iv) prior knee pathologies, including tumors, pseudotumorous lesions, rheumatic autoimmune disorders, and metabolic abnormalities; (v) concomitant pain originating from other knee structures, the hip, or the lumbar spine; and (vi) contraindications to MRI. After applying these criteria, 91 patients were excluded, leaving 215 eligible patients. Orthopedic outpatients (*n* = 182) were randomly assigned to training (*n* = 109) and internal test (*n* = 73) datasets in a 6:4 ratio, with individual differences [age, sex, and body mass index (BMI)] controlled to minimize confounding effects. Patients referred by community general practitioners (*n* = 33) were included in the external testing dataset (Figure 1). Patient data, including height, weight, age, sex, surgery history, trauma, rheumatism, metabolic joint conditions, daily physical activity levels [quantified using the International Physical Activity Questionnaire (IPAQ) scores], and knee joint pain and functional status [quantified using the Anterior Knee Pain Scale (AKPS)], were collected.

### 2.3 MRI acquisition

All MRI procedures were performed using a Magnetom Vida 3 Tesla (Siemens, Erlangen, Germany) machine equipped with an 18-channel knee coil. Following an explanation of the examination precautions, the patients' knees were positioned in slight flexion between 15° and 20°. Cotton cushions were used to stabilize the flexed position of the knee joint and to enhance patient comfort. Initial assessments were conducted using standard MRI sequences, including coronal T1-weighted images (T1WI) and transverse, sagittal, and coronal PDWI. A multi-echo Q-Dixon technique was employed for FF quantification. This method uses multiple echoes to generate accurate fat and water fraction maps, enabling rapid and comprehensive fat quantification within a single breath-hold. Furthermore, the Q-Dixon technique facilitates concurrent evaluation of both FF and transverse relaxation time (T2*). The detailed sequence parameters are provided in Table 1.

**Table 1:** Parameters of the magnetic resonance imaging protocol.

Sequence	TR (ms)	TE (ms)	FA (°)	Section thickness/spacing (mm)	Voxel resolution (mm)	FOV	Acquisition time
Coronal T1WI	363	12	90	3	0.4 × 0.4 × 3.0	160	68 s
Sagittal PDWI	2,240	30	150	3	0.5 × 0.5 × 3.0	160	87 s
Coronal PDWI	2,350	30	150	3	0.5 × 0.5 × 3.0	160	91 s
Transverse PDWI	2,240	30	150	3	0.5 × 0.5 × 3.0	160	87 s
Sagittal liver lab	9	1.05, 2.46, 3.69, 4.92, 6.15, 7.38	4	0.9	1.2 × 1.2 × 1.2	230	35 s

T1WI, T1-weighted image; PDWI, proton density-weighted image; TR, repetition time; TE, echo time; FA, flip angle; FOV, field of view.

### 2.4 MRI assessment

**Figure 2:**
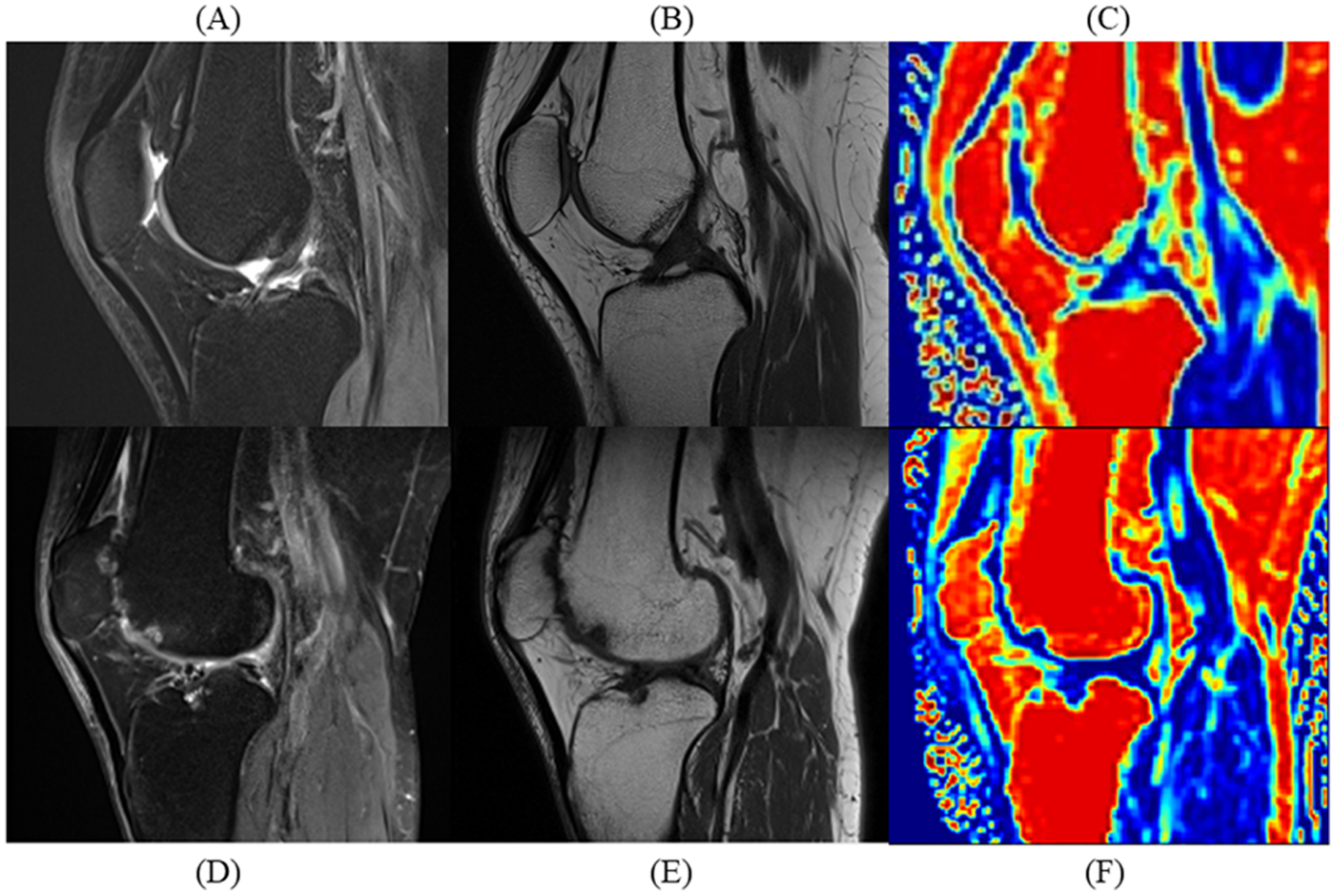
Comparative sagittal MRI scans of patients with and without patellofemoral osteoarthritis (PFOA). Sagittal MRI scans illustrate quadriceps fat pad (QFP) differences between a 49-year-old male without PFOA and a 50-year-old male with severe PFOA. **(A)** Proton density-weighted turbo spin-echo imaging (PDWI) of the PFOA patient without PFOA shows uniform hypointensity in QFP. **(B)** T1-weighted imaging (T1WI) of the patient without PFOA shows uniform fat-like intensity in QFP. **(C)** Fat fraction (FF) mapping of the patient without PFOA showing high-fat content in QFP. **(D)** PDWI of the patient with PFOA showing an elevated signal in QFP. **(E)** T1WI of the patient with PFOA shows hypointensity in QFP. **(F)** FF mapping of the patient with PFOA shows reduced fat content in QFP.

All patients were assigned unique identification numbers, and all MR images were anonymized. Two musculoskeletal radiologists (J.R. and L.J.), with 4 years and 14 years of experience, respectively, assessed each patient's MRI scans using the patellofemoral joint-specific items of the MRI Osteoarthritis Knee Score (MOAKS) through a consensus-based approach. The MOAKS (7, 25) is a commonly used semi-quantitative tool for scoring KOA adapted from the Whole Organ Magnetic Resonance Imaging Score and the Boston Leeds Osteoarthritis Knee Score. This adaptation led to improved convenience and reliability compared with the precursor scoring systems. The MOAKS divides the knee joint into 14 distinct subregions and assigns scores based on the severity of articular cartilage loss, bone marrow lesions, and osteophytes. MRI-PFOA was defined (7, 25) as the presence of both a definite osteophyte (MOAKS-osteophyte score ≥2) and cartilage damage (MOAKS-cartilage size score ≥2 or full-thickness loss score ≥1) within the patellofemoral joint (Figure 2).

### 2.5 MR image processing and segmentation

**Figure 3:**
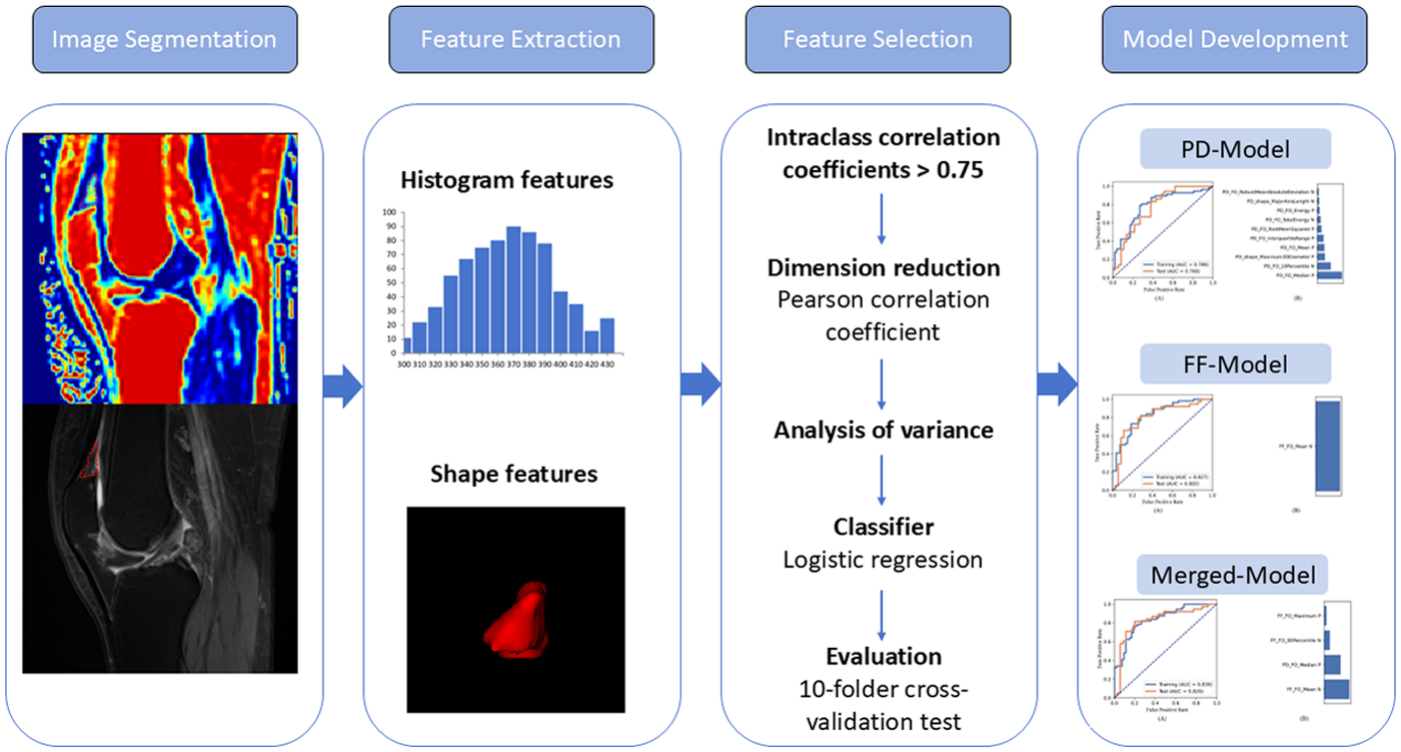
Image-processing pipeline.

The image-processing pipeline is illustrated in Figure 3. First, all images were resampled to a 1 × 1 × 1 mm^3^ isotropic voxel resolution. Following registration, a senior radiologist (L.J.) segmented the QFPs based on anatomical landmarks using the ITK-SNAP program (version 3.6.0) to generate a 3D mask from the sagittal PDWI and FF mapping images. Intra- and inter-observer reliabilities were assessed by randomly selecting 30 patients' images, with J.R. performing the segmentation, followed by repetition three weeks later by two blinded observers (L.J. and J.R.).

### 2.6 Feature extraction, selection, and classification model building

Features were extracted from each segmented volume using FeAture Explorer Pro (FAE, version 0.5.13) in Python (version 3.7.6) following normalization. These features were selected based on our previous study (25). Instead of using high-dimensional and computationally intensive features, first-order features evaluating the distribution of voxel intensity within the QFP on sagittal PDWI/FF maps and shape-related features derived from the original images were extracted for further analysis. A total of 18 first-order features were included: energy, entropy, minimum, maximum, mean, median, range, interquartile range, mean absolute deviation, robust mean absolute deviation, root mean squared, skewness, kurtosis, variance, uniformity, total energy, 10th percentile, and 90th percentile. In addition, 14 shape-related features were analyzed: voxel volume, surface-to-volume ratio, surface area, mesh volume, maximum diameter, minor axis length, elongation, flatness, least axis length, major axis length, maximum 2D diameter column, maximum 2D diameter row, maximum 2D diameter slice, and maximum 3D diameter. To ensure reproducibility and reliability only features with intraclass correlation coefficients (ICCs) greater than 0.75 were selected for further analysis.

Data from orthopedic outpatient and community datasets were independently collected. To balance the distribution of positive and negative samples in the dataset, random upsampling was applied. The feature matrix was normalized by centering each vector through mean subtraction and further normalization based on its magnitude. To address high dimensionality, feature similarities were assessed by calculating Pearson's correlation coefficients. Any pair of features with a correlation coefficient exceeding 0.990 had one member removed to ensure feature independence. This curation step reduced redundancy and enhanced the streamlining of data features for analysis. Subsequently, an analysis of variance (ANOVA) was performed to identify the most relevant features, with F-values calculated to quantify feature-label correlations. Features were ranked by their *F*-values, and the top-ranking features were selected for model construction. Three classification models were developed based on feature sets derived from PDWI (PD model), FF maps (FF model), and a combination of both (merged model). Logistic regression was used as the classifier. To optimize the hyperparameters, such as the number of features included, a 10-fold cross-validation was performed on the training dataset. The optimal hyperparameters were determined by evaluating model performance on the validation dataset. Python (version 3.7.6) and FeAture Explorer Pro (FAE, version 0.5.13) were employed for the entire process.

### 2.7 Statistical analysis

Baseline clinical information was compared using the *t*-test or Mann–Whitney *U*-test for continuous variables and the chi-squared test for categorical variables. Reliability was assessed by calculating the ICC using 30 randomly selected knees. ANOVA and chi-squared tests were employed to evaluate clinical differences between the datasets. Receiver operating characteristic (ROC) curve analysis, along with the area under the ROC curve (AUC), was performed to evaluate model performance. The 95% confidence interval (CI) was estimated using bootstrapping with 1,000 samples to enhance the robustness of the results. The DeLong test was used to compare the AUCs of different models. All statistical analyses were conducted using SPSS software (version 24.0; IBM Corporation, Armonk, NY, USA). A two-sided *p*-value of <0.05 was considered statistically significant.

## 3 Results

### 3.1 Patient characteristics

The mean age of the study cohort was 54.2 ± 11.3 years, and 113 patients were female. The training, internal test, and external test datasets included 109 patients (56/53 positive/negative), 73 patients (38/35 positive/negative), and 33 patients, respectively. Clinical information for each dataset is provided in Table 2. The IPAQ scores were not significantly different across the datasets. However, the AKPS scores showed significant differences, with the training and internal test datasets demonstrating lower scores compared with the external test dataset (80.5 ± 8.5 and 82.1 ± 8.4 vs. 85.1 ± 7.4, *p* = 0.04). There was no missing data, and the details of sample size estimation are provided in Supplementary Materials.

**Table 2:** Clinical characteristics of the patients.

Characteristics	Training set (*n* = 109)	Internal test set (*n* = 73)	External test set (*n* = 33)	*P*-value
Age (mean ± SD)	53.6 ± 10.5	53.8 ± 12.3	57.2 ± 11.5	0.84
Sex (female)	58 (53.2)	38 (52.1)	17 (51.5)	0.99
BMI (kg/m^2^)	24.2 ± 3.8	23.9 ± 3.0	24.9 ± 4.5	0.52
IPAQ	1.7 ± 0.8	1.8 ± 0.3	1.6 ± 0.8	0.09
AKPS	80.5 ± 8.5	82.1 ± 8.4	85.1 ± 7.4	0.04

IPAQ, International Physical Activity Questionnaire; AKPS, anterior knee pain scale; BMI, body mass index; SD, standard deviation.

### 3.2 Reliability of radiomics features

First-order and shape-related QFP features exhibiting excellent intra- and inter-observer reliability (ICC ≥0.75) were included in subsequent analyses (Supplementary Material). There were eight FF-original first-order, six FF-original shape-related, eight PD-original first-order, and six PD-original shape-related features.

### 3.3 Model performance in training and internal test sets

**Figure 4:**
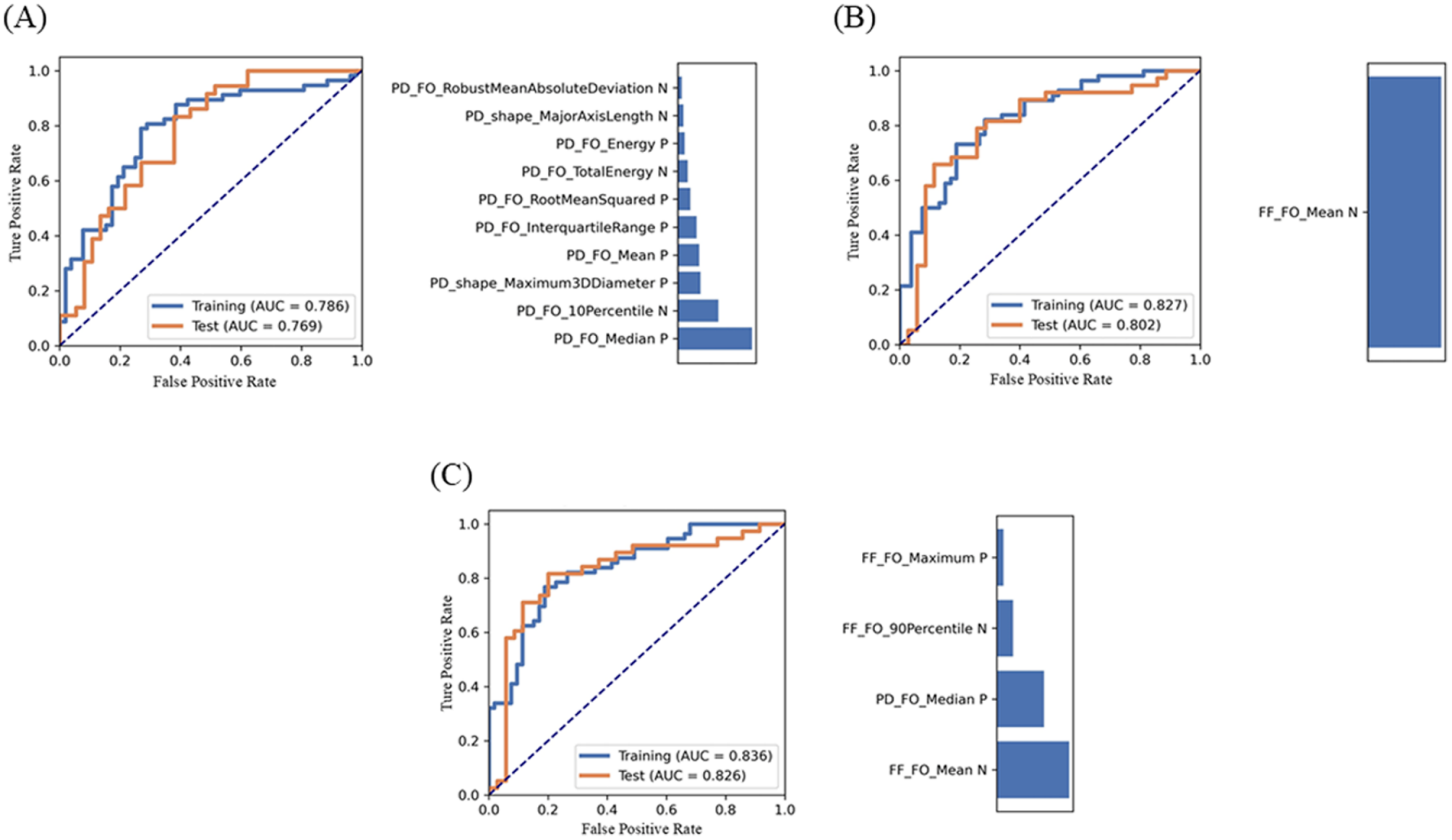
Performance of the PD model **(A)**, the FF model **(B)**, and the merged model **(C)** and their feature contribution of performance metrics generated using feature explorer software. Receiver operating characteristic (ROC) curves illustrate the model's performance in the training and internal test sets. The bar chart shows the contribution of selected features to each model's performance.

The merged model, constructed using the four features, demonstrated the best performance with an AUC of 0.836 (95% CI: 0.762–0.910) in the training set and 0.826 (95% CI: 0.722–0.929) in the internal test set. For the PD model, the AUCs in the training and test sets were 0.786 (95% CI: 0.698–0.874) and 0.769 (95% CI: 0.660–0.876), respectively. The FF model achieved an AUC of 0.827 (95% CI: 0.751–0.903) in the training set and 0.802 (95% CI: 0.694–0.909) in the internal test set (Table 3). Significant differences in the AUC were observed across the training, internal test, and external test datasets (all *p* < 0.001). The ROC curves and feature weights for the models are presented in Figure 4.

**Table 3:** Performance of the radiomics models in the training and test sets.

Cohort	Model	AUC (95% Cl)	Accuracy	Sensitivity	Specificity
Training	PD	0.786 (0.698–0.874)	0.762	0.790	0.731
	FF	0.827 (0.751–0.903)	0.771	0.732	0.811
	Merged	0.836 (0.762–0.910)	0.789	0.768	0.811
Internal test	PD	0.769 (0.660–0.876)	0.726	0.833	0.622
	FF	0.802 (0.694–0.909)	0.767	0.658	0.886
	Merged	0.826 (0.722–0.929)	0.808	0.816	0.800
External test	PD	0.711 (0.532–0.891)	0.636	0.500	0.800
	FF	0.860 (0.734–0.987)	0.727	0.765	0.688
	Merged	0.885 (0.768–1.000)	0.879	0.833	0.933

Cl, confidence interval; AUC, area under the curve; FF, fat fraction.

### 3.4 External test set performance

To further evaluate the optimal model's performance, 33 additional cases from the community were independently assigned to an external test set and analyzed using the same feature selection and classification processes applied to the training and internal test sets. The merged model demonstrated the highest diagnostic performance, achieving an AUC of 0.885 (95% CI: 0.768–1.000) and accuracy, sensitivity, and specificity values of 0.879, 0.833, and 0.933, respectively (Table 3). The FF model also showed strong diagnostic potential, achieving an AUC of 0.860 (95% CI: 0.734–0.987), an accuracy of 0.727, a sensitivity of 0.765, and a specificity of 0.688.

## 4 Discussion

### 4.1 Key findings

This study investigated the potential value of QFP analysis based on conventional PDWI and convenient Q-Dixon sequences for distinguishing PFOA from non-PFOA using machine learning algorithms. The key findings include the following: (i) the merged model, utilizing ANOVA for feature selection and logistic regression for classification, achieved the highest AUC; (ii) the feature “FF_original_firstorder_Mean” extracted from FF mapping exhibited excellent diagnostic performance in differentiating PFOA from non-PFOA; (iii) shape-based features added limited value to differentiation; and (iv) features derived from FF mapping appeared more relevant to PFOA than those from PDWI, with the mean FF value being particularly significant. FF mapping based on the Q-Dixon sequence represents a promising imaging tool for the diagnosis, treatment, and follow-up of OA due to its convenience and quantifiable nature.

### 4.2 Comparison with prior studies

Our findings align with previous studies (26, 27) indicating that women, older adults, and individuals with higher BMIs are more susceptible to PFOA. While the mechanisms underlying sex-specific susceptibility to PFOA are not completely understood, they may be linked to thinner cartilage, increased levels of inflammatory markers, and higher obesity prevalence in females compared with males. An MRI study (28) revealed structural abnormalities in the patellofemoral joint of younger individuals, although these abnormalities did not correlate with AKP. Conversely, other studies (7, 27) have shown that persistent AKP in young to middle-aged adults often presents with radiographic signs of PFOA. Further longitudinal research is needed to determine whether early structural abnormalities of PFOA in younger adults represent a critical target for preventing OA progression. The current study focused on patients aged 40–70 years, minimizing age-related variability. Most of the outpatient population also fell within this range. To control for individual differences, age, sex, and BMI were carefully balanced across the training and testing datasets.

The results revealed no significant differences in IPAQ scores across the datasets, likely owing to the absence of athletes or individuals engaged in manual labor among the study population. However, the AKPS scores significantly differed among the datasets, with patients in the training and internal test sets exhibiting lower AKPS scores than those in the external test set. This disparity may be attributed to the tiered medical treatment system, where patients with more severe symptoms often seek care at larger hospitals rather than community health care centers. Lower AKPS scores indicate decreased functionality and reduced quality of life. Despite being a primary contributor to AKP, PFOA has received less attention than TFJ in OA-related studies. Traditionally, such studies (8, 29) have relied on broad indices, such as the Western Ontario and McMaster Universities Osteoarthritis Index (WOMAC), to measure knee pain. These indices, however, may obscure specific underlying causes of AKP, such as PFOA. To address this limitation, we selected the AKPS as a more precise assessment tool. Further investigation into the pathology underlying AKP symptoms is warranted.

Several studies (14, 30, 31) have validated that quantitative measures of the infrapatellar fat pad (IPFP) can serve as surrogate markers of treatment efficacy in clinical trials on KOA. Notably, changes in FF (32, 33) within the IPFP correlate with OA severity, Hoffa's synovitis, and knee pain, suggesting its potential as an emerging quantitative imaging marker for KOA. Recent radiomics studies (15, 18) have further confirmed the predictive capacity of the IPFP for KOA progression. However, identifying the IPFP can be challenging in some patients because of its irregular and slit-like shape, particularly in the presence of large joint effusions (34). In contrast, the QFP, another peripatellar fat pad, is gaining increased recognition. The QFP is more readily distinguishable on MRI and is less affected by joint effusion. QFP abnormalities, including alterations in signal intensity or morphological changes observed on MRI, have been linked to PFOA and AKP in previous studies (35–37).

In the current study, the QFP, the smallest peripatellar fat pad, exhibited a triangular configuration in sagittal views. Similar to the IFP, the QFP was observed as white fatty tissue with lobes and partitions of uniform size and width. A histological study (38) comparing the suprapatellar and infrapatellar fat pads revealed that the QFP contains smaller adipocytes, while the IFP is rich in type III collagen. Second-harmonic generation microscopy demonstrated an anisotropic collagen distribution in the septa, with the IFP being stiffer, suggesting that anatomical location influences fat pad characteristics. The QFP plays a vital role in facilitating knee movement within the extensor mechanism. Anatomical changes in the QFP can increase pressure and potentially lead to inflammation and hypertrophy, presenting as shape or signal alterations on MRI. Similar to the IFP, the QFP and synovium function as a cohesive unit, contributing to early synovial inflammation, fibrosis, and pain associated with KOA (10, 39). Previous studies (35, 37) have identified correlations between QFP mass effect or signal intensity changes and PFOA, suggesting that shape-related or histogram features of the QFP can serve as important markers for PFOA. Interpatient variability in QFP appearance on MRI is notable. Consequently, this study avoided quantifying the absolute size or gray value of the QFP, focusing instead on shape-related and first-order features derived from radiomics. The findings demonstrated that, in the PDWI model, both shape-related and first-order features contributed to distinguishing PFOA from non-PFOA, reflecting QFP-related imaging changes. Quantitative extraction of shape features offered a more objective definition of the QFP. However, the results also revealed that histogram features were more relevant to PFOA than shape-related features, indicating that inflammatory and metabolic activities within the QFP play a more significant role in PFOA development than morphological changes.

Previous MRI studies (35–37) on QFP relied on traditional PDWI, which provided a semi-quantitative assessment that lacked sufficient sensitivity to pathological alterations, making it suboptimal for planning targeted interventions. In a prior investigation, we identified a relationship between the QFP's FF and T2* values and PFOA severity using the quantitative Q-Dixon technique in patients with AKP (40). Given the sensitivity of T2* to magnetic field homogeneity, we developed three predictive QFP models for PFOA evaluation: the PD model (10 features), the FF model (1 feature), and the merged model (4 features). The merged model, incorporating four features, demonstrated the best diagnostic performance, while the simpler FF model, with only one feature, also showed promising results. This finding suggests that FF derived from Q-Dixon may hold greater clinical significance than traditional gray values derived from PDWI in evaluating PFOA. The merged model integrated three histogram features from FF mapping and one feature from PDWI, with the feature “FF-original first-order Mean” having the most significant impact. This feature demonstrated the strongest correlation with the mean FF value and PFOA severity, making it the most stable and predictive feature in the analysis. This study provides a novel perspective for monitoring QFP changes during PFOA progression. FF mapping of QFP as a quantitative imaging biomarker based on Q-Dixon, along with its radiomic features, offers a promising noninvasive approach for distinguishing between PFOA and non-PFOA cases. This approach has the potential to enhance individualized clinical diagnosis and treatment strategies.

### 4.3 Limitations

This study has several limitations. First, the small external validation sample size of 33 patients may have influenced the comparability of the radiomics model's performance metrics. However, the community referral origin of these patients enhances the generalizability of our conclusions to real-world settings. Future research should include a more extensive and diverse external validation cohort to ensure model robustness and applicability. Second, the use of a single MRI machine, while reducing confounding factors, limits the generalizability of the results. Data collected from standardized MRI machines across multiple centers would provide greater reliability and robustness, helping to validate the model in varied imaging environments. Third, the absence of histopathological examination limits our ability to clarify the relationship between QFP radiomics features and histopathological characteristics. Future studies should integrate histopathological data to strengthen the biological interpretability of QFP imaging findings. Finally, the modest size of this single-center dataset reduces the statistical power and limits the generalizability of the findings. Expanding to multicenter studies with larger, more diverse datasets is essential for a more robust analysis and a comprehensive understanding of the implications of these findings.

### 4.4 Conclusion

Integrating machine learning with radiomics derived from QFP using FF mapping and PDWI or FF mapping alone represents an effective noninvasive approach for distinguishing PFOA from non-PFOA. This approach shows significant promise for enhancing clinical diagnosis and treatment planning. Notably, Q-Dixon-based FF mapping offers a novel avenue for PFOA research, with potential implications for advancing both diagnostic precision and personalized care strategies.

## Data Availability

The raw data supporting the conclusions of this article will be made available by the authors, without undue reservation.
