# Supplementary material for: A machine learning-based radiomics approach for differentiating patellofemoral osteoarthritis from non-patellofemoral osteoarthritis using Q-Dixon MRI
Source: Front Sports Act Living. 2025 Jan 17;7:1535519. doi: 10.3389/fspor.2025.1535519 (PMC11782249; doi:10.3389/fspor.2025.1535519)
Supplement: Supplementary file 1 [file Table1.docx]

**Table. Intra- and inter-observer reliability for the robust features of the quadriceps fat pad (QFP)**

| Category (Setting) | Features | Description | Intra-observer  ICC (≥0.75) | Inter-observer  ICC (≥0.75) |
| --- | --- | --- | --- | --- |
| FF-original first-order | 90 Percentile | 90% of pixels below this value. | 0.99 (0.97–0.99) | 0.96 (0.89–0.99) |
|  | Energy | The magnitude of voxel values | 0.90 (0.77–0.96) | 0.87 (0.65–0.95) |
|  | Maximum | The maximum gray level intensity | 0.99 (0.99–1.00) | 0.98 (0.94–0.99) |
|  | Mean | The average gray level intensity | 0.90 (0.77–0.97) | 0.89 (0.69–0.96) |
|  | Median | The median gray level intensity | 0.93 (0.82–0.97) | 0.91 (0.75–0.97) |
|  | Range | The range of gray values | 0.82 (0.60–0.92) | 0.77 (0.44–0.91) |
|  | Root Mean Squared | The square root of the mean of all the squared intensity values | 0.95 (0.89–0.98) | 0.93 (0.80–0.98) |
|  | Total Energy | The value of Energy feature scaled by the volume of the voxel | 0.93 (0.84–0.97) | 0.90 (0.73–0.97) |
| FF-original shape | Least Axis Length | The least axis length | 0.80 (0.70–0.91) | 0.78 (0.60–0.88) |
|  | Major Axis Length | The largest axis length | 0.89 (0.70–0.92) | 0.85 (0.66–0.89) |
|  | Maximum3D Diameter | The largest pairwise Euclidean distance between surface mesh vertices | 0.89 (0.69–0.94) | 0.84 (0.65–0.90) |
|  | Mesh Volume | The volume calculated from the triangular mesh model | 0.77 (0.51–0.91) | 0.76 (0.45–0.85) |
|  | Surface Area | The total area of the surface | 0.86 (0.69–0.95) | 0.84 (0.70–0.95) |
|  | Voxel Volume | Aggregate volume from voxel quantity. | 0.78 (0.67–0.91) | 0.77 (0.63–0.92) |
| PD-original first-order | 10 Percentile | 10% of pixels below this value. | 0.86 (0.68–0.94) | 0.75 (0.40–0.91) |
|  | Energy | The magnitude of voxel values | 0.86 (0.70–0.94) | 0.80 (0.50–0.93) |
|  | Interquartile Range | The difference between third and first quartiles | 0.81 (0.58–0.92) | 0.76 (0.43–0.91) |
|  | Mean | The average gray level intensity | 0.92 (0.82–0.97) | 0.85 (0.62–0.95) |
|  | Median | The median gray level intensity | 0.99 (0.96–0.99) | 0.95 (0.86–0.98) |
|  | Robust Mean Absolute Deviation | The mean distance of all intensity values | 0.80 (0.68–0.93) | 0.78 (0.46–0.92) |
|  | Root Mean Squared | The square-root of the mean of all the squared intensity values | 0.79 (0.55–0.92) | 0.75 (0.41–0.91) |
|  | Total Energy | The value of Energy feature scaled by the volume of the voxel | 0.86 (0.69–0.95) | 0.82 (0.54–0.94) |
| PD-original shape | Least Axis Length | The least axis length | 0.80 (0.70–0.91) | 0.78 (0.60–0.88) |
|  | Major Axis Length | The largest axis length | 0.89 (0.70–0.92) | 0.85 (0.66–0.89) |
|  | Maximum 3D Diameter | The largest pairwise Euclidean distance between surface mesh vertices | 0.89 (0.69–0.94) | 0.84 (0.65–0.90) |
|  | Mesh Volume | The volume calculated from the triangular mesh model | 0.77 (0.51–0.91) | 0.76 (0.45–0.85) |
|  | Surface Area | The total area of the surface | 0.86 (0.69–0.95) | 0.84 (0.70–0.95) |
|  | Voxel Volume | Aggregate volume from voxel quantity. | 0.78 (0.67–0.91) | 0.77 (0.63–0.92) |

Note: Data are presented as intraclass correlation coefficients (95% confidence intervals). ICC: intraclass correlation coefficient.

**Sample size**

Among our three models, the PD Model, which has the highest number of features, incorporated 10 features. Adhering to the principle that the sample size should be 10 to 15 times the number of features, our acquired sample size of 215 should be adequate. Concerns about overfitting were mitigated by external validation, which demonstrated that the model also performs well in external settings. This suggests that potential issues arising from the small sample size may not be as severe as initially suspected, addressing this concern to a certain extent.
